# Measuring the impact of gene prediction on gene loss estimates in Eukaryotes by quantifying falsely inferred absences

**DOI:** 10.1371/journal.pcbi.1007301

**Published:** 2019-08-28

**Authors:** Eva S. Deutekom, Julian Vosseberg, Teunis J. P. van Dam, Berend Snel

**Affiliations:** Theoretical Biology and Bioinformatics, Department of Biology, Science faculty, Utrecht University, Utrecht, The Netherlands; CPERI, GREECE

## Abstract

In recent years it became clear that in eukaryotic genome evolution gene loss is prevalent over gene gain. However, the absence of genes in an annotated genome is not always equivalent to the loss of genes. Due to sequencing issues, or incorrect gene prediction, genes can be falsely inferred as absent. This implies that loss estimates are overestimated and, more generally, that falsely inferred absences impact genomic comparative studies. However, reliable estimates of how prevalent this issue is are lacking. Here we quantified the impact of gene prediction on gene loss estimates in eukaryotes by analysing 209 phylogenetically diverse eukaryotic organisms and comparing their predicted proteomes to that of their respective six-frame translated genomes. We observe that 4.61% of domains per species were falsely inferred to be absent for Pfam domains predicted to have been present in the last eukaryotic common ancestor. Between phylogenetically different categories this estimate varies substantially: for clade-specific loss (ancestral loss) we found 1.30% and for species-specific loss 16.88% to be falsely inferred as absent. For BUSCO 1-to-1 orthologous families, 18.30% were falsely inferred to be absent. Finally, we showed that falsely inferred absences indeed impact loss estimates, with the number of losses decreasing by 11.78%. Our work strengthens the increasing number of studies showing that gene loss is an important factor in eukaryotic genome evolution. However, while we demonstrate that on average inferring gene absences from predicted proteomes is reliable, caution is warranted when inferring species-specific absences.

## Author summary

To understand the evolution of eukaryotic species, we can look at the differences and similarities in their genomes. Since the first genomes were sequenced, scientists have, among other things, been studying these differences and similarities by evaluating the presences and absences of genes, and they have been trying to understand how these patterns explain the evolution of different eukaryotic species from their last common ancestor. It is now known that the evolution from the last eukaryotic common ancestor was dominated by gene loss and duplications. Here we want to take the presence and absences patterns of genes in 209 diverse eukaryotic species as a guideline to estimate the loss of genes in these different species after they evolved from their common ancestor. Following this, we want to quantify how this loss estimate and the inferred absences of genes are influenced by faulty gene predictions by comparing absences of predicted proteins and absences of genes in genomes. A difference in these absences will indicate that some of them are not absent at all. It is important to quantify how many genes are falsely inferred as absent due to prediction problems and if this can be estimated from certain suspicious patterns in absences. Our results show that overall gene absences are inferred reliably. However, suspicious absences in a species, i.e. absences that are species specific and not supported by absences in other closely related species, have a higher chance of being falsely inferred.

## Introduction

During the evolution of eukaryotic genomes, the number of gene loss events is estimated to be higher than gene gains [1–4] and this high loss gives rise to patchy phylogenetic patterns of gene occurrence. A high level of gene loss suggests a gene rich ancestor of eukaryotes. Alternatively, patchy phylogenetic patterns of genes could also be indicative of horizontal gene transfer (HGT) from prokaryotes to eukaryotes or from eukaryotes to eukaryotes [3]. Nevertheless, studies showed that generally these patchy patterns are better explained by differential gene loss and gene presence in the Last Eukaryotic Common Ancestor (LECA) [1,3] and not by HGT. It has been proposed that in evolution new genes and functional repertoires originate in rapid genome expansions, followed by adaptive genome streamlining, or gene loss, giving rise to divergent species [5,6].

A small number of highly debated reports on gene losses [7,8] turned out to have incorrectly inferred genes as lost [9–11]. In fact, there are many reasons to presume that not all inferences of gene loss are equally trustworthy. The number of genomes published that do not exceed draft quality is increasing, resulting in annotation errors and errors in the number of genes found in the genome [12]. This suggests that the reported high number of loss events to some extent could result from genes whose absences have been falsely inferred. Genes can be inferred as absent for multiple reasons: due to technical difficulties in genome sequencing [10], due to misassembly of draft genomes, due to faulty protein prediction [12], due to insensitivity/bias in sequence similarity detection [13], or they are a bona fide loss. Recently, partial Pfam domain hits were in part attributed to incomplete gene models, yet another type of gene prediction and annotation problem [14].

Measuring the absences of genes that are expected to be universally conserved in organisms is a popular measure of genome annotation quality and completeness. The CEGMA pipeline [15] and later the BUSCO tool [16] successfully implemented this principle using near-universal single-copy orthologs. Absences of these single-copy orthologs are considered to be suspect and are widely used to quantitatively assess annotation quality and genome completeness.

While analysing the kinetochore protein complex and the absences of its subunits in eukaryotes, we recently showed that 10.9% of these absences could be found in six-frame translated DNA [17]. These falsely inferred absences in the kinetochore included important subcomplexes that would have otherwise been assumed to be absent in multiple species. One example was KNL1, a two sub-unit complex consisting of Knl1 and Zwint1, which plays a crucial role in microtubule attachment to the centromeres during mitosis. The KNL1 complex was wrongly inferred as completely or partially absent in 19 out of 109 species due to prediction problems. For 3 out of 19 species the complete complex was incorrectly inferred as absent, for 5 species the subunit Knl1 was falsely inferred as absent, and for 11 species the subunit Zwint1 was falsely inferred as absent. The study also showed that absences have a higher chance of being falsely inferred when they were species-specific absences or made little biological sense due to e.g. functional restrictions in protein complexes [17].

There is ample anecdotal evidence that poor gene annotation will influence gene loss analyses [7,17]. However, we here aim to systematically quantify the impact of gene prediction on the estimated gene loss by reanalysing absences inferred from predicted proteomes by analysing six-frame translated DNA. In particular, we hypothesize that absences that are not supported by absence in sister taxa are more likely to be false. Therefore, additional to the overall analysis of absences, we test the hypothesis that species-specific absences will be more likely falsely inferred as absent. We find that gene prediction in general is trustworthy and that loss remains an evolutionary important factor in eukaryotic genome evolution, with the caveat that suspicious, or species-specific, absences have a substantially higher chance of being falsely inferred.

## Results

### Loss of inferred ancestral Pfams

To measure the impact of gene prediction on apparent gene loss in eukaryotes, we first inferred a list of proteins that indicate loss of these proteins in present-day species. For this we first estimated their presence in the Last Eukaryotic Common ancestor (LECA). Instead of utilizing orthologous relations, we used the Pfam domain family database [13] to detect homologous protein domains in the predicted proteomes of present-day species. Pfam domains have the advantage that they are clearly defined units for detecting protein homology, whereas other databases would make it necessary to differentiate between paralogs and orthologs of partial hits in the DNA or make it necessary to call fusion and fission relationships of genes, which is easily subject to error and remains one of the largest problems within bioinformatics [18]. Another advantage of using Pfam is that it allows us to compare our loss and LECA estimates to previous work that analysed eukaryotic genome evolution on the scale of protein domains [1].

We analysed the presence of Pfam domains in 209 proteomes from a diverse set of eukaryotic species that can be divided into six supergroups: Amoebozoa, Archeaplastida, Cryptophyta/Haptophycea, Excavata, Opisthokonta and SAR (consensus species tree shown in S1 Fig and species summarized in S1 Table). We then inferred potential LECA domain presences using the Dollo parsimony method and consequently inferred losses. In this method, domains can only be gained once and domain losses are minimised. The resulting LECA domain content consists of 5479 Pfams (Table 1, Proteome data), which is comparable to the LECA content of Pfam domains as previously estimated by [1] using a similar method. Our estimate of LECA content is higher than the previously estimated LECA content (5479 versus 4431) as we use more species, as well as more evolutionary distant species.

**Table 1:** Summary of data and results from the proteome and six-frame translated genomes.

	**Proteome data (N = 209)**		
	**BUSCOs**	**Pfams**	
		**Non-strict LECA**^a^	**Strict LECA**^b^
**LECA domains**	303	5479	4182
**Total count domains**	47874	1145111	874038
**Species-specific absences**	5791	97655	71559
**Clade-specific absences**	n/a	419323	218203
**Absences**	6055	516978	289762
**Loss**	-	162671	111320
**Median loss**		30	26
	**Six-frame translated genome data**		
	**BUSCOs (N = 158)**^c^	**Pfams (N = 199)**^c^	
**Found species-specific absences**	1093	-	13111
**% found species-specific absences**	18.87%		18.95%
**Median % found species-specific absences**	18.30%	-	16.88%
**Found clade-specific absences**	n/a	-	4301
**% found clade-specific absences**	n/a	-	2.05%
**Median % found clade-specific absences**	n/a	-	1.30%
**Found total absences**	1093	-	17412
**% found total absences**	18.87%		6.24%
**Median % found total absences**	18.30%		4.61%
**Loss**	n/a	-	98209
**Median loss**	n/a		23

^(a)^ LECA inferred with non-strict Dollo parsimony criteria, similar as [1].

^(b)^ LECA inferred with stricter Dollo parsimony criteria that includes removed horizontal gene transfers.

^(c)^ Only genomes with more than 5 BUSCO absences were added for further calculations, leaving 158 genomes. Due to unforeseen tool crashes during six-frame translation, 199 genomes were left for analysis with the Pfam set.

The LECA gene content inferred from naïve Dollo parsimony is very sensitive to horizontal gene transfers (HGT). If there were independent HGT events from bacteria to multiple lineages at both sides of the root in the eukaryotic tree, the Dollo parsimony method would incorrectly infer presence of that domain in LECA and thus infer many incorrect loss events. Therefore, we subsequently removed Pfam domains that were likely HGTs from bacteria to increase the reliability of our LECA estimate. These possible HGT Pfams were inferred based on a phylogenetic position of eukaryotic sequences among prokaryotic sequences or on being present in a small subset of eukaryotic species. Upon removal of these Pfams, the LECA content decreased to 4182 Pfams (Table 1, Proteome data. Pfams shown in S2 Table). The 4182 LECA domains were inferred to be lost 111320 times, with a median of 26 losses per domain in our set of 209 species (Table 1). Our results are in line with previous reports, which also find a large number of gene loss [1–3].

### Quantifying falsely inferred absences and possible differences between clade- and species-specific absences

**Fig 1:**
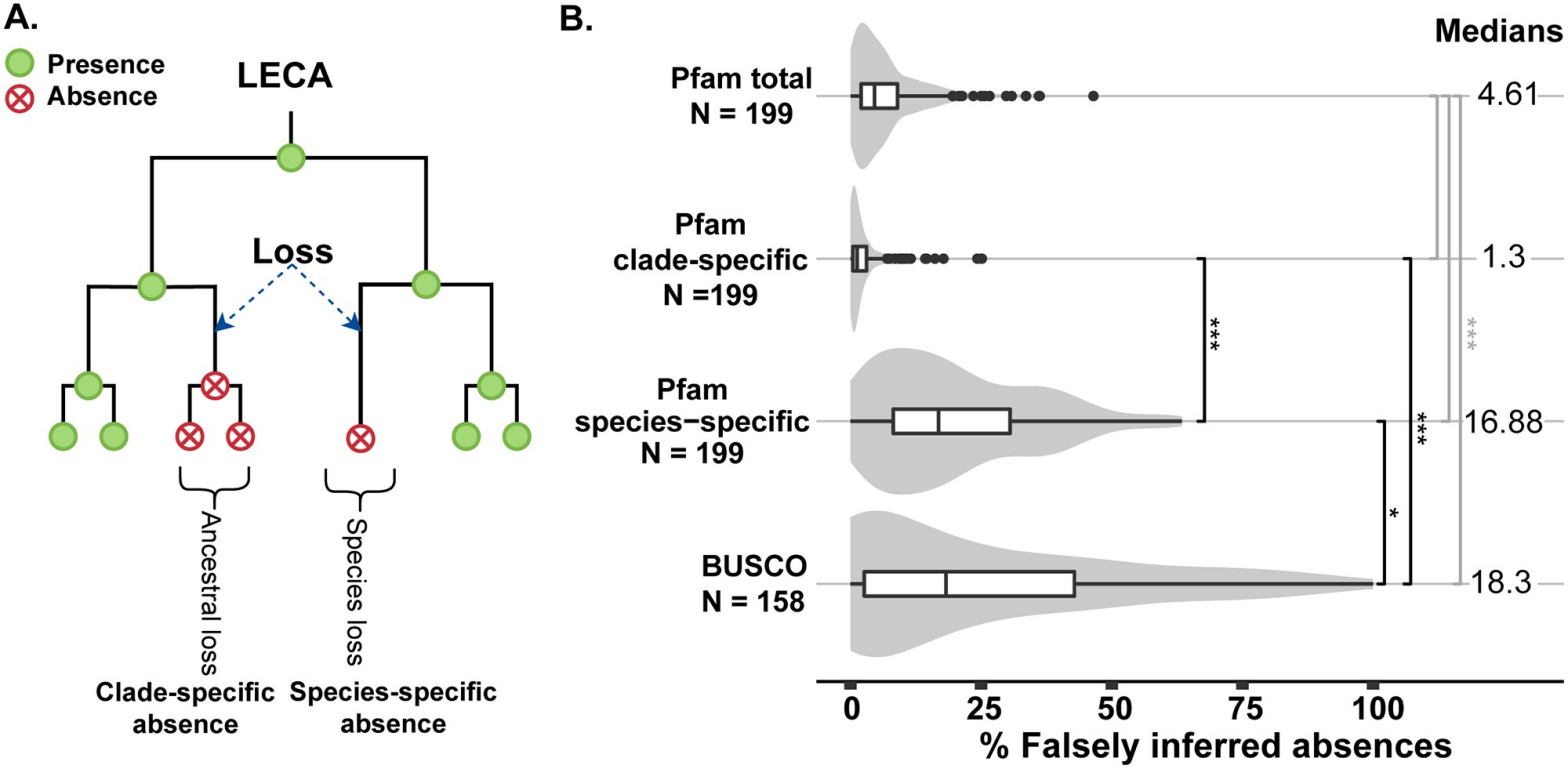
False inference of different absences. (A) Graphical representation of two different types of absences and loss. Clade-specific absences are phylogenetically supported by an ancestral loss. Neighbouring species, i.e. the clade, have the same absence. Species-specific absences are not phylogenetically supported by an ancestral loss, or in other words it is a single loss. A loss is independent of previous losses, in other words the first time a gene is lost. (B) Percentages of falsely inferred absences in different absence groups across genomes. From top to bottom the violin plots show: the percentages of falsely inferred absences in the total Pfam set absences, clade-specific absences and species-specific absences, and the BUSCO set absences. Since the BUSCO set contains a small number of domains (303), only the genomes with more than five absences (N = 158) were added to this figure. Note that the Pfam results are based on 199 species (N = 199) due unforeseen tool crashes during the analysis (see Materials and methods and S1 Table). Significance levels of pairwise comparisons between groups are given with black asterisks and comparisons between total absences and the rest of the groups in grey. Significance levels are *** for p ≤ 0.001 and * for p ≤ 0.05 (Wilcoxon signed rank test). Data is summarized in Table 1. Violin plots are scaled to have the same maximum width.

An absence might be falsely inferred as a loss due to sequencing issues, genome assembly issues or incorrect gene prediction. While we are unable to correct for the sequencing and assembly issues, we are able to identify possible falsely inferred absences. We performed a hmmsearch of LECA Pfam domains against six-frame translated genomes of the proteomes that were initially analysed. Two examples of falsely inferred absences are schematically shown in S2 Fig. Not all Pfam hits were expected to be true presences, since the residual homology of pseudogenes can also lead to the detection of a Pfam. Therefore, we excluded hits containing stop codons in the alignments as they are likely pseudogenes and instead inferred an absence. Following this, our pipeline retrieved hits for 6.24% of all previously inferred Pfam absences (17412), which thus represent potentially falsely inferred absences (Table 1), with a median of 4.61% over all our 199 six-frame translated genomes (“Pfam total” in Fig 1B). This estimate provides an upper estimate for this problem and as shown below is largely driven by a specific subset of false absences.

Previous analyses suggested that not all absences were equally likely to be correct [17]. Absences that made little biological sense, i.e. were suspicious, tend to have a higher chance of being falsely inferred as absent. Often a suspicious loss was an observed single absence in a single species amidst a larger clade. To explore if there is a difference in detecting falsely inferred absences between suspicious and non-suspicious absences, we defined two categories of absences: clade-specific and species-specific (Fig 1A). Clade-specific absences are supported by an ancestral loss, meaning they are supported by absences in one or more directly related species with independently sequenced and annotated genomes. Species-specific absences are not supported by losses in directly related species. Absences in the BUSCO domain set (see Introduction) can be classified as suspicious absences as well, since all eukaryotes are assumed to have these single-copy orthologs. BUSCO therefore functions as an additional independently derived measurement of species-specific absences. We quantified to what extent these two types of absences are falsely inferred.

We found significant differences between species-specific and clade-specific absences in terms of their likelihood to be found in six-frame translated DNA. We found hits for 18.95% of the species-specific absences (Table 1), with a median of 16.88% per genome (Fig 1B). We found hits for 18.87% of the BUSCO absences, with a median of 18.30% per genome (Table 1 and Fig 1B). The median of falsely inferred species-specific absences in the Pfam set is surprisingly similar to that of the BUSCO absences, despite a weak positive correlation between these two sets (S3 Fig). In contrast, we found substantially (and significantly) lower hit percentages for clade-specific absences, with only 2.05% found for the clade-specific absences, with a median of 1.30% per genome. This 10-fold difference between found clade- and species-specific absences demonstrates that a species-specific absence has a higher chance to be a false absence than an absence that is supported by sister lineages. Moreover, it is this high rate of falsely inferred species-specific absences which significantly raises the overall rate of found absences to 6.24%.

Additionally, we focussed more on the phylum taxonomic level to see if there is a change in falsely inferred absences when looking at different phyla (S4 Fig). We observe the same trend as that on the level of LECA, with species-specific and BUSCO absences being more falsely inferred as absent than clade-specific absences. There seems to be no specific trends in the individual phyla. However, it clearly shows that certain phyla are overrepresented. These results provide a straightforward, but effective way of guiding the detection of possible falsely inferred absences in both large- and small-scale evolutionary analyses.

**Fig 2:**
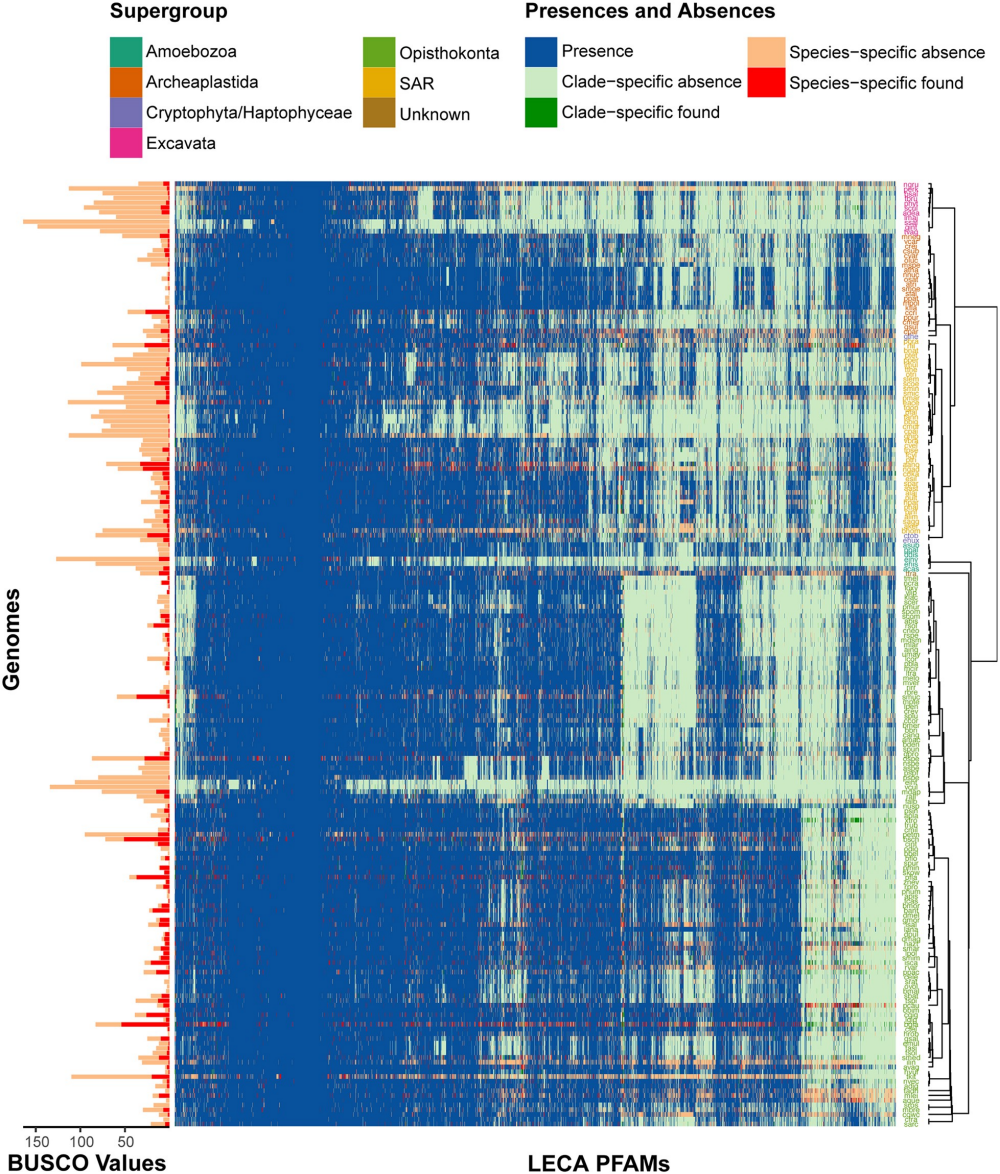
Presences and absences of all LECA Pfams in all 199 species. The barchart (top) shows the BUSCO absences and found BUSCO absences. The large matrix shows presences and all types of absences as shown in the coloured legend. Species are clustered according to the species tree (S1 Fig) shown by the dendrogram. Pfams are clustered with hierarchical (complete-linkage) clustering. Pfam labels are left out for clarity.

From Fig 1B it is also clear that several species have a higher percentage of falsely inferred absences, shown by the outliers (black points). For these species, this could signify that they have either lesser quality genomes or predicted proteomes. In Fig 2 this is highlighted by high instances of red in a particular genome, which indicates a high number of found species-specific absences, or dark green, which indicates a high number of found clade-specific absences. This is also highlighted by the number of BUSCO absences found for the same genome (bar chart Fig 2). The genome specific values depicted in Fig 2 can be found in S1 Table.

We also took a subset of genomes, that can be considered model organisms for evolutionary studies, to analyse if any methodological differences between model and non-model organisms have an effect on falsely inferred absences (S5 Fig). For this subset of model organisms (N = 35), we can observe the same trend as that of the whole dataset, with species-specific absences being more falsely inferred as absent. Surprisingly, for the BUSCO set (N = 21) the median of falsely inferred absences per genome lies higher in the model organism subset, 34.29% compared to 18.30%. Additionally, looking at N50 values of all the genomes, a proxy for genome assembly quality, we can see no significant link between falsely inferred absences and N50 values (S6 Fig). Therefore, rather unexpectedly, it appears that completeness of sequencing or assembly problems are not an indication for higher expected false absences.

Another effect did become apparent during the analysis: short Pfam domains have a higher chance to be falsely inferred as absent. S7 Fig shows Pfam lengths of the top 100 highest numbers of falsely inferred Pfam absences, showing a significant difference (almost twice as much) between the median of the Pfam lengths of the 100 most found Pfam absences versus the rest. This trend is potentially explained by short single domain proteins that fall just below the commonly used cut-off length of 100 amino acids in genome annotation pipelines for proteins with only *in silico* evidence [19,20].

### Impact of incorrect gene prediction on gene loss estimates in eukaryotes

**Fig 3:**
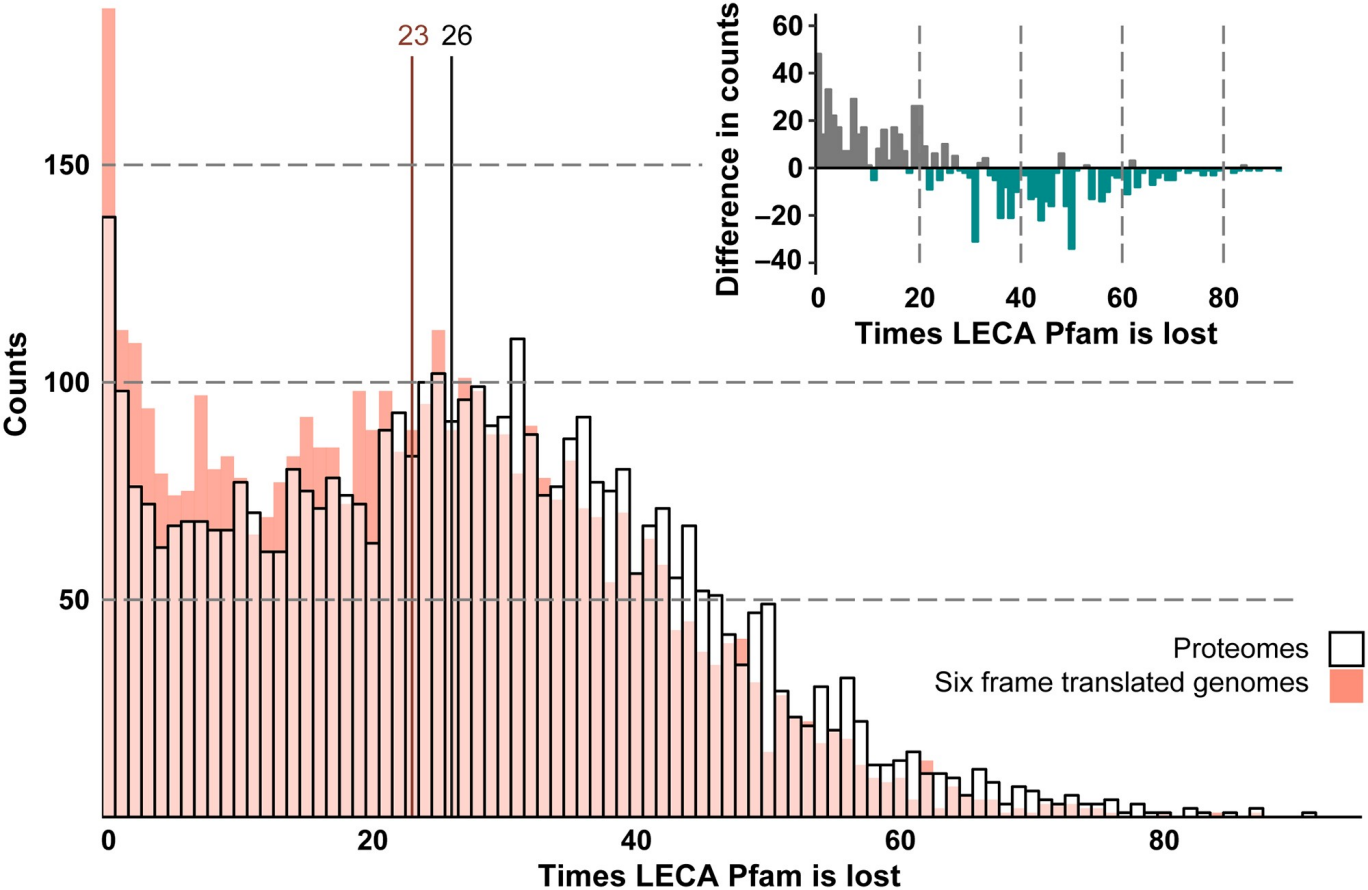
Distribution of the estimated loss of LECA Pfam domains in proteomes shown by white bars, with the median loss given by the black vertical line. The Dollo parsimony approach places 4182 Pfams in LECA. These LECA Pfams have been lost independently 111320 times. A large number of Pfams are conserved in all current day species (never lost). Distributions of the corrected loss of LECA Pfam domains from six-frame translated genomes are shown by orange coloured bars, with the corrected median loss given by the red vertical line. The inset shows the difference in distributions of the six-frame translated genomes minus the proteomes.

To answer the question whether incorrect gene prediction could influence genome evolution inferences, we re-analysed the loss events and corrected our initial estimated domains loss by including the hits we found in six-frame translated DNA. Fig 3 shows the loss corrected with the Pfam domains found in six-frame translated genomes (coloured bars) and the uncorrected loss according to proteomes (white bars). The number of times a LECA Pfam is lost in general shifts to lower values (Fig 3 inset). The number of Pfams with many loss events decreased. Notably, the Pfam domains that were conserved in all species, i.e. lost zero times, showed the largest increase, from 138 to 186 domains.

The found hits decrease the amount of loss by 11.78%, from 111320 to 98209, reducing the median loss per Pfam from 26 to 23 (p-value < 2.2⋅10^−16^ Wilcoxon signed rank test) (Table 1 and Fig 3 vertical lines in histogram). The reason for this relatively higher impact on loss estimates, despite the smaller percentage of 6.24% falsely inferred absences, is that every species-specific loss is counted equally as a clade-specific loss (Fig 1A). Since species-specific absences are much more likely to be falsely inferred as absent than clade-specific absences, they have relatively more impact on the amount of loss. Thus, species-specific loss and their higher likelihood for being falsely inferred as absent is a significant issue in comparative genomics studies on gene loss.

## Discussion

Eukaryotic genome evolution is dominated by gene duplication and gene loss [1–4,6]. However, absences of genes in predicted proteomes do not always indicate that these genes are truly lost. During the past few years high profile reports of specific cases of gene loss (peptide hormone ghrelin in soft-shell and sea turtle [7] and multiple genes in birds [8]) were disproven [9–11]. Falsely inferred absences could greatly influence conclusions drawn when analysing genome evolution, the evolutionary trajectory of proteins or protein complexes and adaptation of organisms. In our study, we showed that per genome 4.61% of absences are falsely inferred to be absent. Additionally, we showed that for the two different types of absences these percentages differ significantly: clade-specific absences are only falsely inferred as absent 1.30% of the time, but species-specific are falsely inferred as absent 16.88% (Pfam) and 18.30% (BUSCO) of the time.

Our estimates rely on the specific design of our analysis, such as the use of Pfam HMMs and the Dollo parsimony approach. The Dollo parsimony approach is a simplified way of describing eukaryotic genome evolution: only one domain gain is allowed and the number of losses is minimized, effectively ignoring HGT events. The importance of HGT in eukaryotes remains controversial and is still an active area of study [21, 22]. Nevertheless, the usage of Dollo parsimony allows direct comparisons with a similar approach previously described in Zmasek & Godzik [1], as well as give a straightforward way of defining LECA for identifying patterns in absences and identifying clade- and species-specific absences. Even though we are not trying to infer the gene content of LECA, we want to estimate the LECA Pfam content as accurate as possible because otherwise we cannot reliably interpret absences in terms of loss. Therefore, we additionally added a stricter criterion for accepting Pfams as LECA Pfams and removed possible HGT using a phylogenomics approach.

Our combined Dollo parsimony and phylogenetic HGT filtering approach, yields a LECA size in terms of Pfam domains comparable to that of Zmasek & Godzik [1] and in terms of genes to that of Wolf & Koonin et al. [6]. It would be expected that the increased sampling in our work of more diverse genomes, such as free-living heterotrophs and poorly sampled taxa, would increase the number of inferred LECA Pfams compared to these earlier studies. At the same time, the phylogenetic approach for removing suspected HGT families, decreases the number of inferred LECA Pfams.

The number of losses might be influenced by uncertainties in the tree of life and its topology: a clade-specific absence might become a species-specific absence and vice versa due to minor rearrangements in the used tree topology. However, we expect that this will not significantly influence the results, since in general the leaves of the tree are confidently assigned and the uncertainties often lie in the specific hierarchy in higher-level taxonomy, such as the location of the root of the eukaryotic tree of life [23–25].

It is important to note that over the years improvements in species sampling and sensitivity in homology detection have led to drastically expand the gene content of LECA and in turn increase in loss events to the high numbers now reported [1–4,6]. However, the gene prediction problem is not the only technical issue influencing gene loss estimates. Other (technical) issues could also artificially increase gene loss estimates. For example, domain profiles (HMMs) can be insufficiently sensitive due to biased/limited sequence sampling or due to strict bit score cut-offs chosen [13] due to an (understandable) focus on avoiding false positives. Especially in lineages with rapidly evolving genes, unrecognized homologs can be the cause of falsely inferred absences and consequently higher loss estimates. Improving the sensitivity of HMMs of protein domains has anecdotally been shown to improve domain detection [17]. Another issue is incomplete genome assemblies, which preclude genes from being found. For instance, many gene absences in bird genomes were shown to stem from genome assemblies with stretches of strongly decreased coverage due to GC-rich regions [10]. Genes that are falsely inferred as absent due to incomplete sequencing of certain genomes can also not be found by simply searching the DNA sequence for homologs, as is done here. The combined effect of all these issues in addition to gene prediction is not known yet, but could further lower gene loss estimates.

With this study, we want to provide some guiding estimates of the extent of one particular technical problem, i.e. unpredicted genes present in genome sequences. This problem is in practice known, but to our knowledge has never been systematically quantified. Our results show that in general gene prediction is of good quality and inferred absences are likely not false. However, there is more than a 10-fold difference between the number of falsely inferred clade-specific absences (1.30%) versus species-specific absences (16.88% for Pfam and 18.30% for BUSCO). This is directly in line with the observation that ghrelin was already reported in the red-eared slider turtles and later indeed correctly inferred to be present (and not absent) in the genome of soft-shell and sea turtle [9]. The importance of gene loss for eukaryotic molecular evolution is fundamentally not impacted by falsely inferred absences and remains a dominant factor in shaping eukaryotic gene repertoires. Still, loss decreases by 11.78% due to falsely inferred absences that can be found in six-frame translated DNA and our study clearly demonstrates that biologically suspicious absences should invite additional technical scrutiny.

### Conclusion

The results of our study show that when absences are surprising and/or suspicious they have a higher chance of being falsely inferred as absent. This result is especially important for the evolutionary analyses of proteins and their domains and estimating their loss. It provides a cautionary tale that if an absence appears suspicious there is a good reason to investigate this further and conclusions should not only rely on automated gene prediction alone.

Our findings agree with existing notions of gene prediction problems, but no study as of yet has quantified to what extent gene prediction influence gene loss estimates. Our simple but effective approach described in this study provides a straightforward way to analyse gene absences and quickly assess their reliability in large- and small-scale evolutionary analyses.

## Materials and methods

To measure the impact of gene prediction on gene loss estimates we first needed to establish gene content in the last eukaryotic common ancestor (LECA) to infer loss patterns from LECA to current day species. We did this by analysing the presences and absences of protein domains in current day species, and then inferring LECA content using these presence/absences profiles. With this LECA content we inferred the loss of LECA domains in current day species. Following this, we looked at protein domains that are not found in the proteomes to see if they were encoded in the genomes of the respective species. S8 Fig schematically shows the procedures and the following sections describe these procedures in more detail.

### Compiling the database

To study the presences and absences of genes across the eukaryotic tree of life we used predicted proteomes and genomes of 209 phylogenetically diverse eukaryotic organisms from multiple supergroups: 122 Opisthokonta, 6 Amoebozoa, 23 Archaeplastida, 3 Crypto-/Haptophyceae, 13 Excavata, 41 SAR, and 1 unidentified (species summarized in S1 Table). We chose these species to represent a broad eukaryotic diversity. The predicted proteomes and genomes were obtained from a variety of sources (S1 Table).

To examine if absences in the proteomes could still be found in the genomes of their respective species, we used the tool Transeq (Translate nucleic acid sequences) from EMBOSS [26] to translate the genomes in six open reading frames to protein sequences with the default codon table. For ciliate species, we used the ciliate codon table (translation table 6). We successfully analysed 199 of the 209 genomes (S1 Fig). One genome (human) could not be translated due to its large size and transeq crashing as a consequence, two species did not have available genomes and seven translated genomes could not be analysed due to an unknown error in the hmmsearch tool (S1 Table).

### Protein domain content in proteomes and translated genomes

The protein domain repertoire was determined with the hmmsearch alignment tool from the HMMER package 3.1b2 (dated February 2015) [27] using sequence profiles, HMMs (Hidden Markov Models), from the Pfam 31.0 database [28] and the BUSCO eukaryota database (*odb9*) [29]. We took HMM specific quality scores for Pfam (gathering cut-offs) and BUSCO domains to validate the hits in the alignments.

Some Pfam domains could be absent from predicted proteomes because they are (part of) a non-functional gene, i.e. a pseudogene. We therefore removed pseudogenes from our hits in six-frame translated genomes with a custom-built script that removed hits with stop-codons in their sequences. Best scoring non-overlapping hits were considered for further analysis in presence/absence profiles.

### Approximating domain content of the last eukaryotic common ancestor with Dollo parsimony

We used the Dollo parsimony approach for the ancestral state reconstruction, i.e. the domain content of LECA, using presence/absence profiles of Pfam domains in the predicted proteomes and projecting them on a bifurcating species tree. The species tree is a consensus tree combined from literature, which is summarized in S1 File and the species tree shown in S1 Fig). The Dollo parsimony code was updated and translated to python from [30]. This approach allows for a gene/domain to be gained only once through a phylogenetic tree, which may require an arbitrary number of subsequent losses, and traces presences/absences back to the root (LECA) of the tree. We added additional criteria to increase the accuracy of our LECA estimate by only considering Pfam domains that are present in at least 3 supergroups and are left and right of the root (S1 Fig).

To remove Pfams that are in LECA due to possible horizontal gene transfer (HGT), we used a phylogenomics based approach. We inferred and analysed phylogenetic trees based on Pfam sequences containing sufficient phylogenetic signal from a diverse set of prokaryotes and eukaryote to identify possible HGT Pfams as follows. The eukaryotic database described above was supplemented with the prokaryotic proteomes in eggNOG4.5 [31] and the Asgard archaeal predicted proteomes from [32]. Pfam domains were detected with hmmsearch as described above. Reduction of the number of sequences was necessary to make it computationally feasible to apply sequence alignment and phylogenetic reconstruction. To reduce the number of sequences to be used in phylogenetic inference, kClust 1.0 [33] (clustering threshold 2.93) was performed on the eggNOG prokaryotic sequences and a ScrollSaw-like method [2] was applied to the eukaryotic sequences. The sequences in bidirectional best BLAST 2.6.0+ [34] hits (BBHs) between sequences from different sides of the eukaryotic root were selected. For each Pfam the selected prokaryotic and eukaryotic sequences were aligned (mafft v7.310 [35] auto option); these alignments were trimmed (trimAl v1.4.rev15 [36] gap threshold 10%). Phylogenetic trees were inferred with IQ-TREE 1.6.4 [37] (LG4X model, 1000 ultrafast bootstraps [38]). The resulting trees were analysed using the ETE3 toolkit [39].

For each monophyletic eukaryotic clade in a tree, it was first checked if there were species from both sides of the eukaryotic root present in that clade. If at least one such potential LECA clade was present in the tree, the information from the eukaryotic sequences not in the BBHs, and therefore not in the tree, was incorporated. By assigning these sequences to their best representing hit in the tree, the percentage of species in which a homolog from that clade was present was calculated for five supergroups: Excavata, SAR + Haptista, Archaeplastida + Cryptista, Amoebozoa and Opisthokonta + Apusozoa. If the mean of these percentages was at least 15%, the clade was annotated as a LECA clade. If there was at least one LECA clade in a tree, the Pfam was annotated as present in LECA. Having a set of trusted LECA Pfams allowed us to remove the non LECA Pfams resulting from horizontal gene transfer, contamination or the chloroplast endosymbiosis from our LECA set.

We also defined two different groups of absences, clade- and species-specific. Clade-specific absences are supported by an ancestral loss of a domain, while species-specific absences are not (see Fig 1). We analysed events in the leaves of Pfam domain trees generated by Dollo parsimony. Leaves with ancestral losses (Pfam loss in parent node) are defined as clade-specific absences. Leaves with single (independent) losses (Pfam present in parent node) are defined as species-specific losses.

## Supporting information

S1 Fig Species tree. A phylogenetic tree of the species used in this analysis. Supergroups are given indicated the legend and the full names that belong to the abbreviations can be found in S1 Table. Species with asterisks were used to estimate the LECA Pfam content with Dollo parsimony, but for multiple reasons (e.g. no genome available) they could not be used to quantify falsely inferred absences (see Results, Materials & methods and S1 Table).
(PDF)

S2 Fig Example of two hits falsely inferred absences in *Aureococcus anophagefference*. Two Pfam domains previously inferred as absent are found in six frame translated DNA. Shown are the HMM overlapping with the scaffolds (x-axis) together with the bitscore. The hmmsearch tool “sequence output” is shown between the HMM and scaffold.
(PDF)

S3 Fig Percentages of found species-specific Pfam absences vs. BUSCO absences per genome. We fitted a linear model (black line), shown in the graph with a 95% confidence interval (shaded area).
(PDF)

S4 Fig Percentages falsely inferred absences found per genome, grouped per phylum. For all the genomes containing a phylum taxonomic annotation (N = 152), the genomes were grouped per phylum in a bar chart, showing percentages falsely inferred absences coloured by four absence groups. Median values are given by the red points (unless there is only one genome the red point is equal to the result) and for clarity grey dotted lines show 50% falsely inferred values. Individual phyla can highly differ in the number of genomes sampled, with Arthropoda having the highest number.
(PDF)

S5 Fig Percentages falsely inferred absences found in model organisms. Percentages of falsely inferred absences in different absence groups in a subset of genomes representing model organisms (N = 35). The BUSCO set contains a small number of domains (303), only the genomes with more than five absences (N = 21) were added to this figure. Significance levels of pairwise comparisons between groups are given with black asterisks Significance levels are *** for p ≤ 0.001 and * for p ≤ 0.05 (Wilcoxon signed rank test).
(PDF)

S6 Fig Comparing found falsely inferred absences with genome assembly quality (N50). The different panels show the different absence groups versus log(N50) values. In the upper left corner of every panel the correlation coefficient τ is shown and corresponding p-value (Kendall rank correlation). There is little association found between the two values in either of the categories of falsely inferred absences.
(PDF)

S7 Fig Pfam hmm lengths of found absences. Hmm lengths are compared in three different absence groups: all, clade- and species-specific, for the 100 highest numbers of absences vs. the rest. Medians are shown at the top of the graph and significance (Wilcoxon rank sum test) is shown above the comparisons.
(PDF)

S8 Fig The workflow for quantifying falsely inferred absences. The BUSCO data is given in yellow, the Pfam data is given in green and processes are given in blue.
(PDF)

S1 Table Per species data. Information on the species used in this study, including taxonomic information of each species, counts of absences and found absences, and download locations of genomes/proteomes.
(XLSX)

S2 Table Per Pfam data. Information on the inferred LECA Pfams, including lengths and counts of absences and found absences.
(XLSX)

S1 File Species tree resources. The file contains a list of resources used for the reconstruction of the species tree (see S1 Fig). The tree is used in this analysis to project presences (and absences) in the Dollo parsimony approach.
(PDF)
