# Supplementary figures and images for: Measuring the impact of gene prediction on gene loss estimates in Eukaryotes by quantifying falsely inferred absences
Source: PLoS Comput Biol. 2019 Aug 28;15(8):e1007301. doi: 10.1371/journal.pcbi.1007301 (PMC6736253; doi:10.1371/journal.pcbi.1007301)

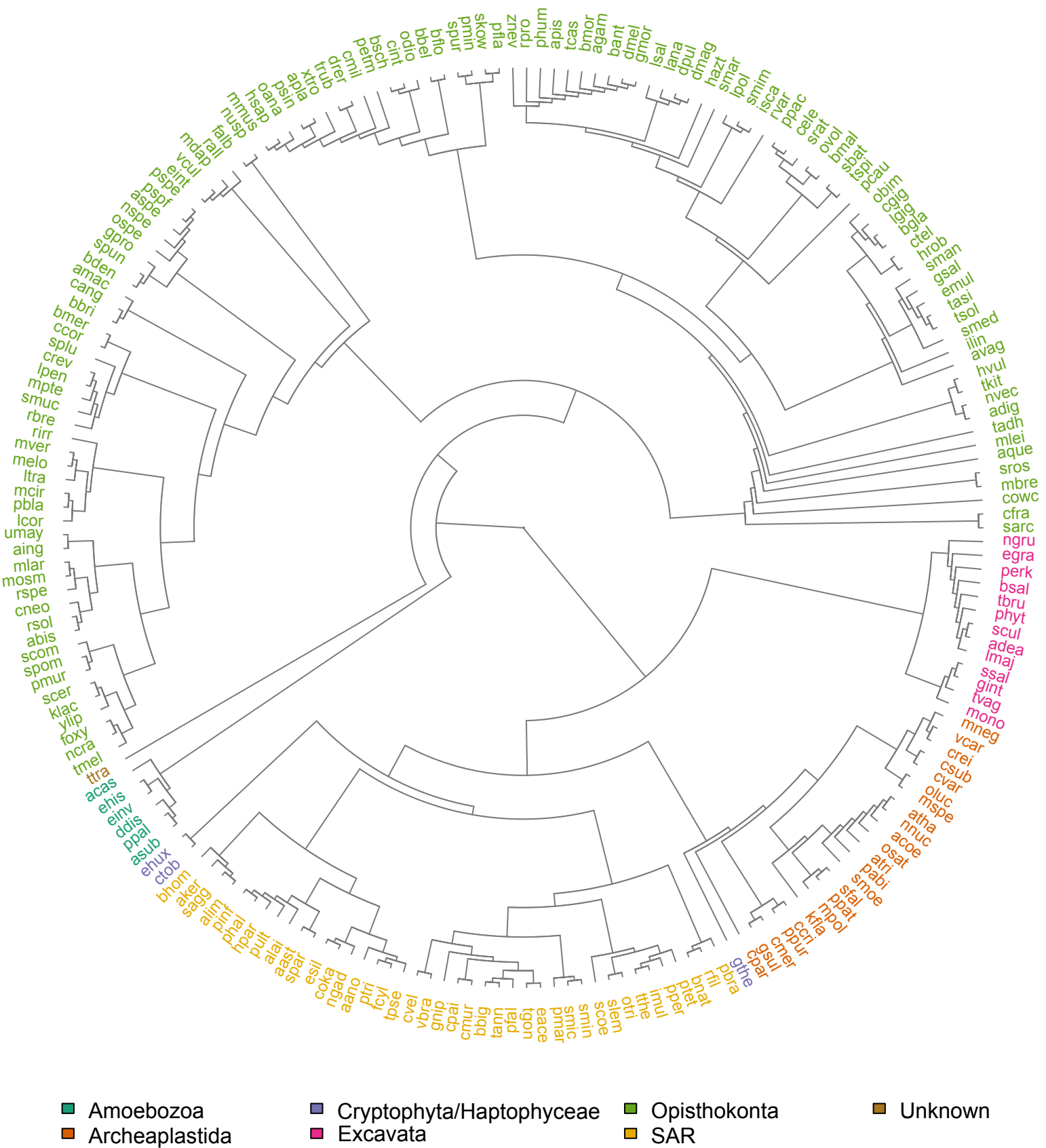

Supplement: S1 Fig — A phylogenetic tree of the species used in this analysis. Supergroups are given indicated the legend and the full names that belong to the abbreviations can be found in S1 Table. Species with asterisks were used to estimate the LECA Pfam content with Dollo parsimony, but for multiple reasons (e.g. no genome available) they could not be used to quantify falsely inferred absences (see Results, Materials & methods and S1 Table). (PDF) [file pcbi.1007301.s001.pdf]

% Found species-specific absences Pfam

$$y = 11.075 + 0.403 x$$

$$R^2 = 0.508$$

$$p < 2.2e-16$$

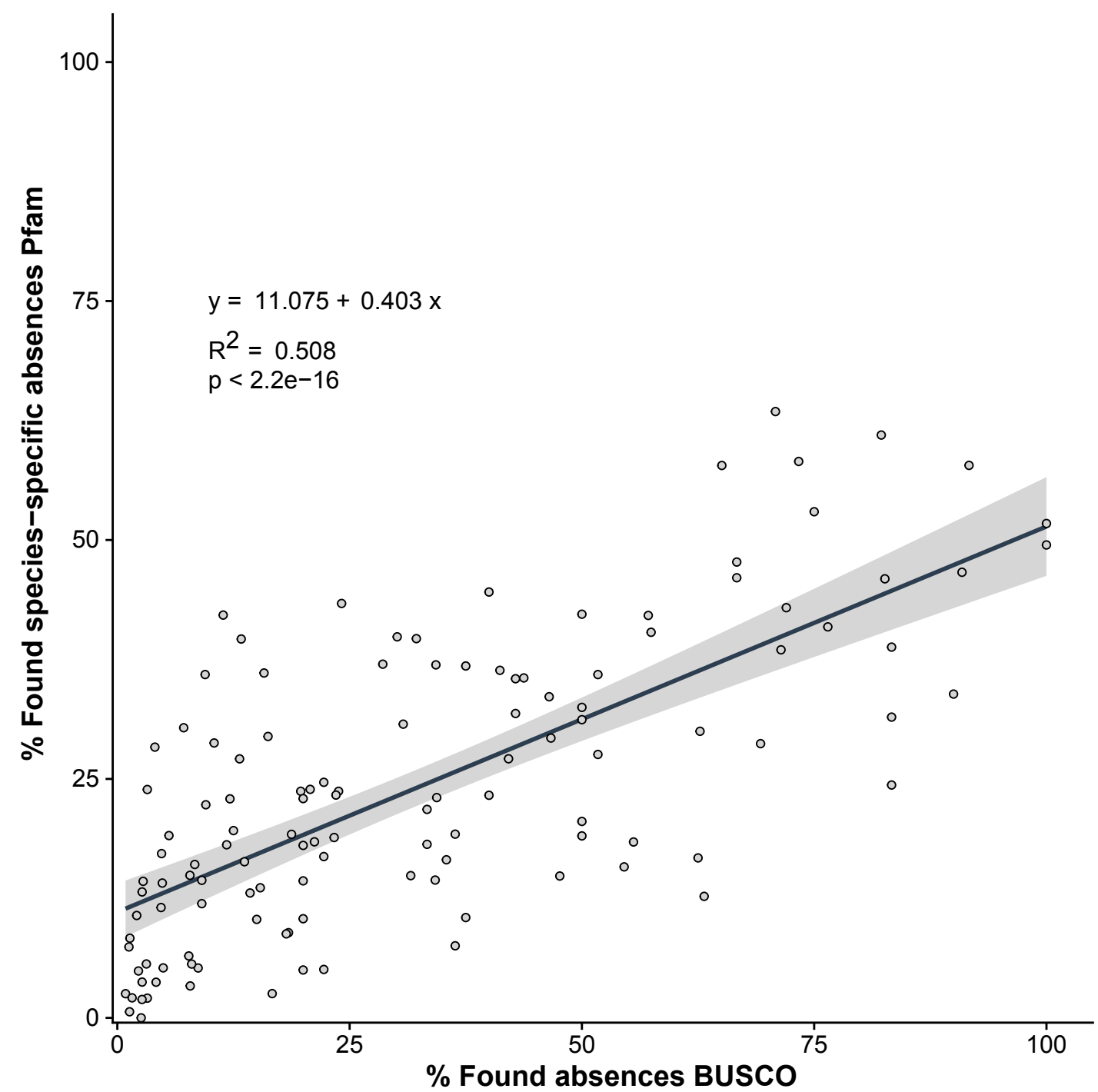

Supplement: S3 Fig — We fitted a linear model (black line), shown in the graph with a 95% confidence interval (shaded area). (PDF) [file pcbi.1007301.s003.pdf]

Taxonomic level: phylum

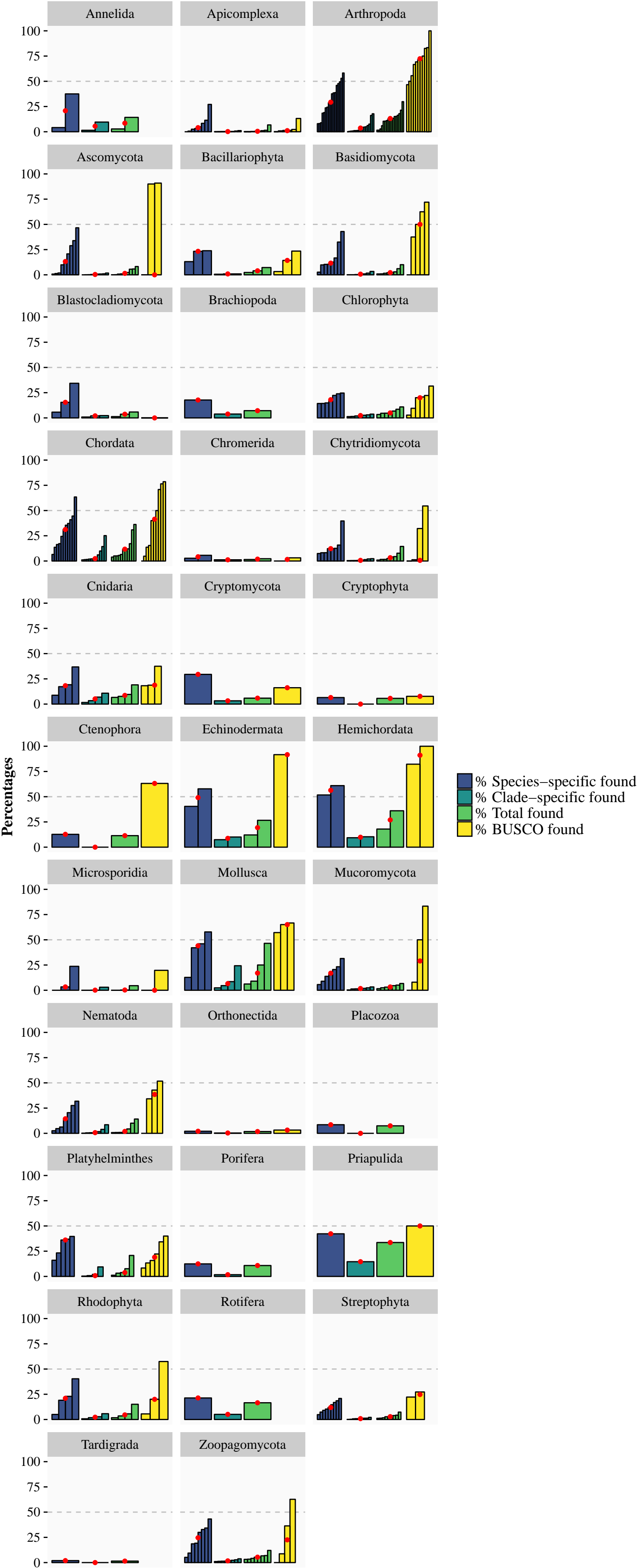

Supplement: S4 Fig — For all the genomes containing a phylum taxonomic annotation (N = 152), the genomes were grouped per phylum in a bar chart, showing percentages falsely inferred absences coloured by four absence groups. Median values are given by the red points (unless there is only one genome the red point is equal to the result) and for clarity grey dotted lines show 50% falsely inferred values. Individual phyla can highly differ in the number of genomes sampled, with Arthropoda having the highest number. (PDF) [file pcbi.1007301.s004.pdf]

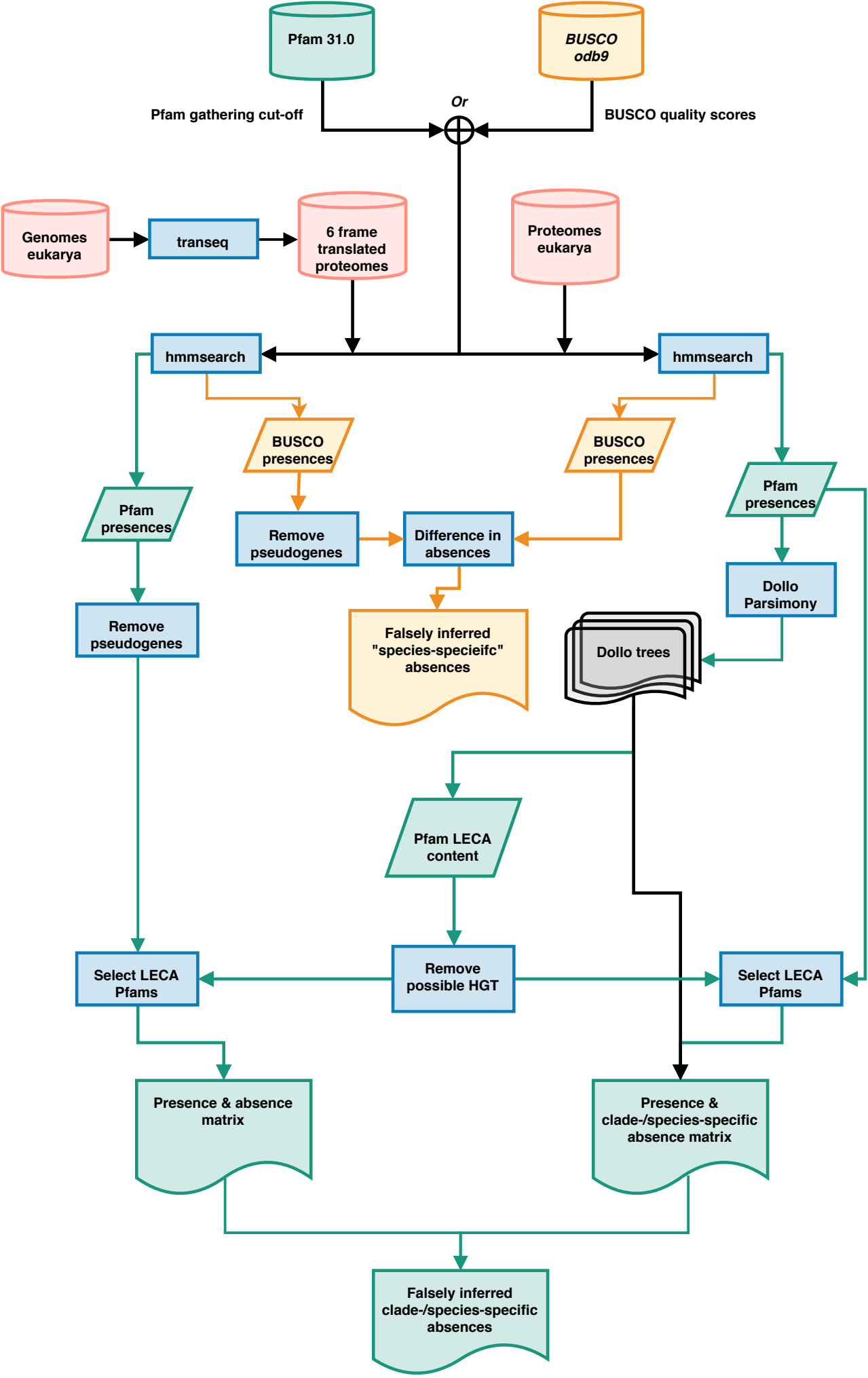

Supplement: S8 Fig — The BUSCO data is given in yellow, the Pfam data is given in green and processes are given in blue. (PDF) [file pcbi.1007301.s008.pdf]
