# Supplementary material for: Measuring the impact of gene prediction on gene loss estimates in Eukaryotes by quantifying falsely inferred absences
Source: PLoS Comput Biol. 2019 Aug 28;15(8):e1007301. doi: 10.1371/journal.pcbi.1007301 (PMC6736253; doi:10.1371/journal.pcbi.1007301)
Supplement: S5 Fig — Percentages of falsely inferred absences in different absence groups in a subset of genomes representing model organisms (N = 35). The BUSCO set contains a small number of domains (303), only the genomes with more than five absences (N = 21) were added to this figure. Significance levels of pairwise comparisons between groups are given with black asterisks Significance levels are *** for p ≤ 0.001 and * for p ≤ 0.05 (Wilcoxon signed rank test). (PDF) [file pcbi.1007301.s005.pdf]

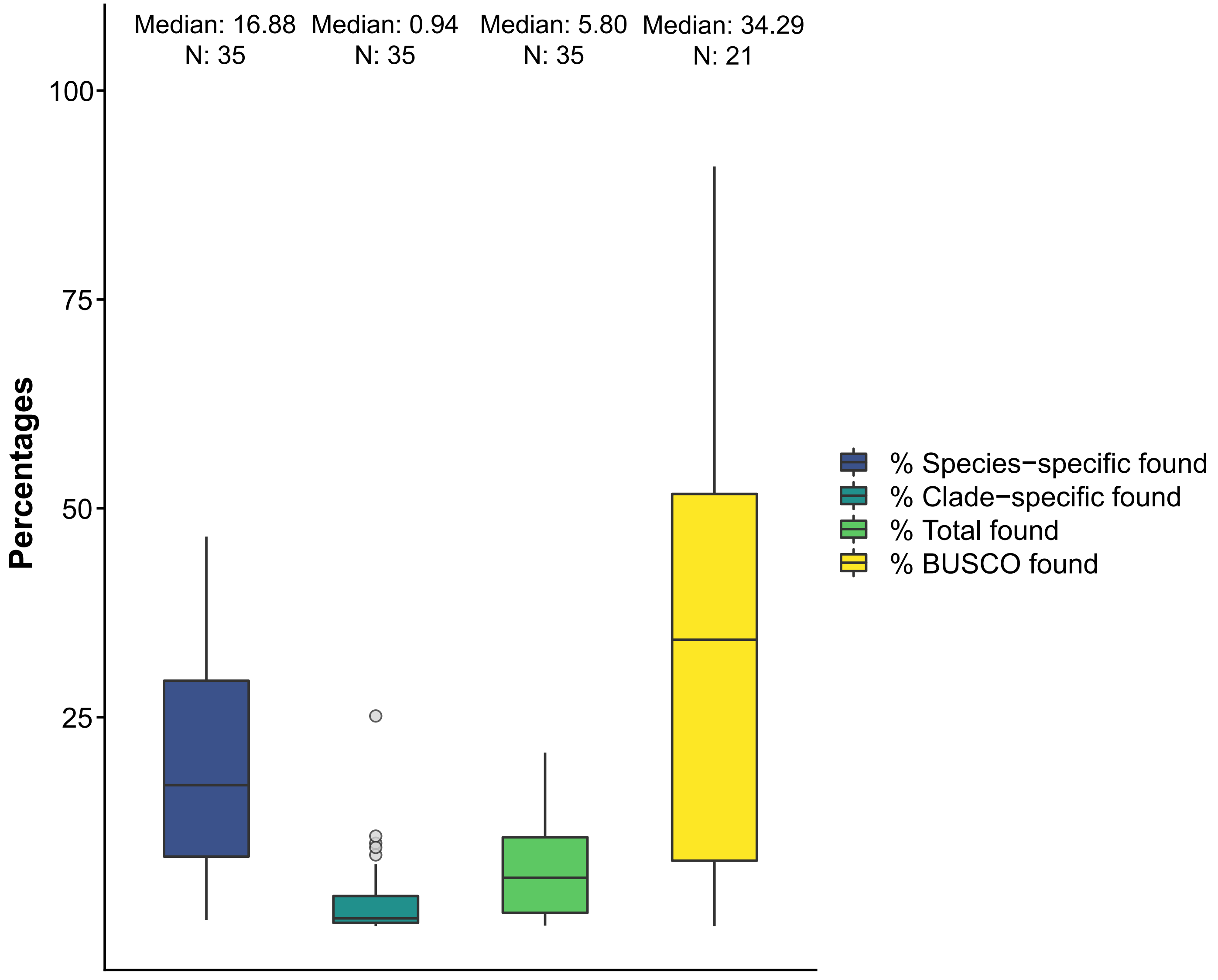

Median: 16.88
N: 35
Median: 0.94
N: 35
Median: 5.80
N: 35
Median: 34.29
N: 21
100
75
50
25
Percentages
% Species−specific found
% Clade−specific found
% Total found
% BUSCO found
