# Supplementary material for: Measuring the impact of gene prediction on gene loss estimates in Eukaryotes by quantifying falsely inferred absences
Source: PLoS Comput Biol. 2019 Aug 28;15(8):e1007301. doi: 10.1371/journal.pcbi.1007301 (PMC6736253; doi:10.1371/journal.pcbi.1007301)
Supplement: S6 Fig — The different panels show the different absence groups versus log(N50) values. In the upper left corner of every panel the correlation coefficient τ is shown and corresponding p-value (Kendall rank correlation). There is little association found between the two values in either of the categories of falsely inferred absences. (PDF) [file pcbi.1007301.s006.pdf]

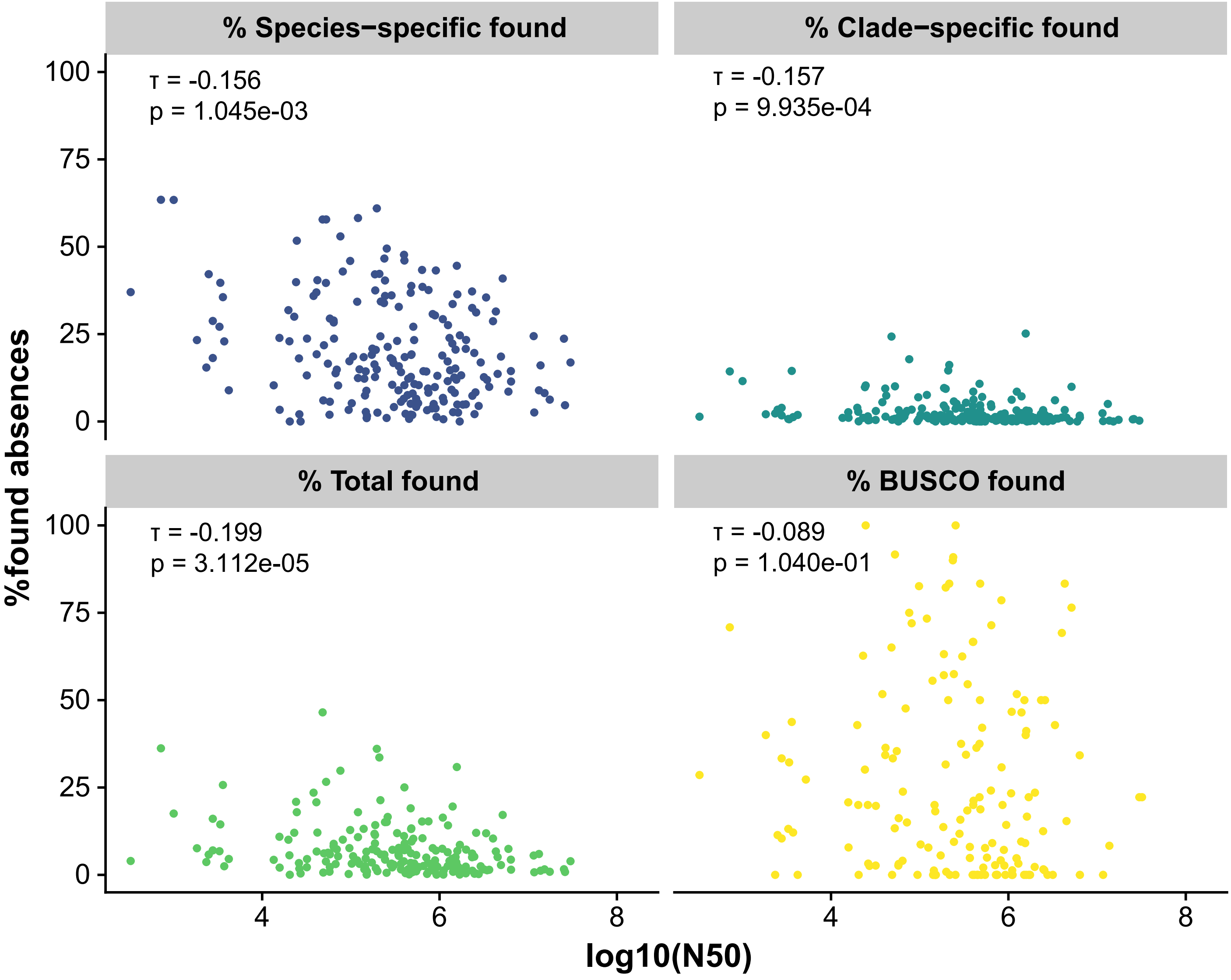

% Species−specific found
% Clade−specific found
% Total found
% BUSCO found
τ = -0.156
p = 1.045e-03
τ = -0.157
p = 9.935e-04
τ = -0.199
p = 3.112e-05
τ = -0.089
p = 1.040e-01
%found absences
log10(N50)
0
25
50
75
100
4
6
8
