# Supplementary material for: Measuring the impact of gene prediction on gene loss estimates in Eukaryotes by quantifying falsely inferred absences
Source: PLoS Comput Biol. 2019 Aug 28;15(8):e1007301. doi: 10.1371/journal.pcbi.1007301 (PMC6736253; doi:10.1371/journal.pcbi.1007301)
Supplement: S7 Fig — Hmm lengths are compared in three different absence groups: all, clade- and species-specific, for the 100 highest numbers of absences vs. the rest. Medians are shown at the top of the graph and significance (Wilcoxon rank sum test) is shown above the comparisons. (PDF) [file pcbi.1007301.s007.pdf]

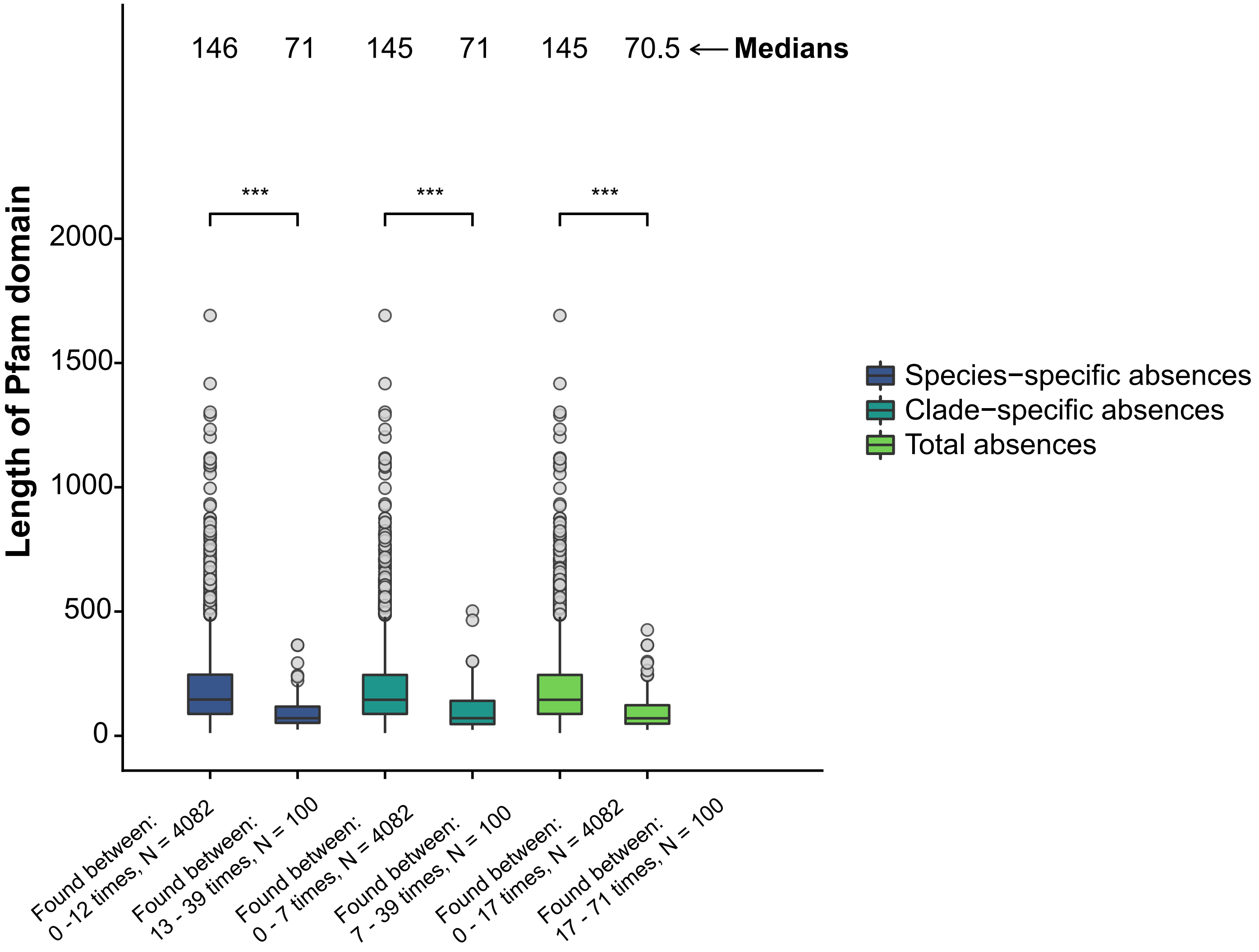

146
71
145
71
145
70.5
Medians
***
***
***
Length of Pfam domain
2000
1500
1000
500
0
Species−specific absences
Clade−specific absences
Total absences
Found between: 0 -12 times, N = 4082
Found between: 13 - 39 times, N = 100
Found between: 0 - 7 times, N = 4082
Found between: 7 - 39 times, N = 100
Found between: 0 - 17 times, N = 4082
Found between: 17 - 71 times, N = 100
