# Supplementary material for: Measuring the impact of gene prediction on gene loss estimates in Eukaryotes by quantifying falsely inferred absences
Source: PLoS Comput Biol. 2019 Aug 28;15(8):e1007301. doi: 10.1371/journal.pcbi.1007301 (PMC6736253; doi:10.1371/journal.pcbi.1007301)
Supplement: S1 File — The file contains a list of resources used for the reconstruction of the species tree (see S1 Fig). The tree is used in this analysis to project presences (and absences) in the Dollo parsimony approach. (PDF) [file pcbi.1007301.s011.pdf]

Supergroups	Resources
Eukaryota	http://www.pnas.org/content/112/7/E693.full<br>http://rspb.royalsocietypublishing.org/content/283/1823/20152802.long?trendmd-shared=0 (contains 'Centrohelida' and MMETSP data)
Opisthokonta	http://www.sciencedirect.com/science/article/pii/S0960982215008878<br>http://www.sciencedirect.com/science/article/pii/S1055790314002796<br>http://www.sciencedirect.com/science/article/pii/S0960982217301999<br>http://www.pnas.org/content/112/48/14912.full<br>https://elifesciences.org/content/4/e05503<br>http://www.nature.com/nature/journal/v477/n7365/full/nature10382.html<br>http://rspb.royalsocietypublishing.org/content/276/1660/1245<br>http://www.sciencedirect.com/science/article/pii/S0960982215007952<br>http://www.sciencedirect.com/science/article/pii/S0960982213000298<br>https://www.nature.com/ng/journal/v36/n12/full/ng1472.html<br>http://science.sciencemag.org/content/346/6210/763.full<br>https://bmcevolbiol.biomedcentral.com/articles/10.1186/1471-2148-13-5<br>http://www.ingentaconnect.com/content/ima/imafung/2010/00000001/00000002/art00020<br>http://www.ingentaconnect.com/content/nhn/pimj/2013/00000030/00000001/art00008<br>http://www.umich.edu/~mycology/resources/Publications/Spatafora.Mycologia.2016.pdf<br>http://jcm.asm.org/content/50/1/66.abstract<br>https://academic.oup.com/mbe/article/26/1/27/972479/Phylogenomic-Analyses-Support-the-Monophyly-of#77767571<br>http://www.sciencedirect.com/science/article/pii/S143446101200079X<br>http://www.nature.com/nature/journal/v443/n7113/full/nature05110.html<br>https://www.nature.com/articles/ncomms5471#
Amoebozoa	http://www.sciencedirect.com/science/article/pii/S1055790314002784<br>http://www.sciencedirect.com/science/article/pii/S105579031630015X<br>http://www.sciencedirect.com/science/article/pii/S1434461012000922<br>http://www.sciencedirect.com/science/article/pii/S1055790314002796<br>http://www.sciencedirect.com/science/article/pii/S1055790315001608<br>https://bmcgenomics.biomedcentral.com/articles/10.1186/s12864-015-1278-x<br>https://academic.oup.com/mbe/article/doi/10.1093/molbev/msx162/3827454/Between-a-Pod-and-a-Hard-Test-The-Deep-Evolution
Archaeplastida	http://currents.plos.org/treeoflife/article/red-algal-phylogenomics-provides-a-robust-framework-for-inferring-evolution-of-key-metabolic-pathways/<br>http://currents.plos.org/treeoflife/article/red-algal-phylogenomics-provides-a-

	robust-framework-for-inferring-evolution-of-key-metabolic-pathways/<br>http://www.pnas.org/content/111/45/E4859.full<br>http://www.sciencedirect.com/science/article/pii/S0960982210014600<br>http://rstb.royalsocietypublishing.org/content/372/1713/20150509<br>http://www.tandfonline.com/doi/full/10.1080/07352689.2011.615705?scroll=top&needAccess=true
SAR	http://mbe.oxfordjournals.org/content/33/11/2890.full<br>http://mbe.oxfordjournals.org/content/33/4/980.full<br>http://www.sciencedirect.com/science/article/pii/S1055790316300677<br>http://msphere.asm.org/content/2/2/e00095-17<br>http://tolweb.org/Apicomplexa<br>http://www.pnas.org/content/114/2/E171.full (mainly dinoflagellates)<br>https://www.ncbi.nlm.nih.gov/pubmed/28417959<br>https://bmcgenomics.biomedcentral.com/articles/10.1186/s12864-015-1904-7<br>https://www.ncbi.nlm.nih.gov/pmc/articles/PMC5394536/<br>https://www.nature.com/articles/srep36089<br>https://www.nature.com/articles/srep24874?WT.feed_name=subjects_taxonomy<br>http://aem.asm.org/content/74/3/883.full<br>http://onlinelibrary.wiley.com/doi/10.1111/j.1550-7408.2008.00379.x/full
Excavata	http://www.pnas.org/content/106/10/3859.full<br>http://www.scielo.br/scielo.php?pid=S0074-02762016001200765&script=sci_arttext<br>https://www.ncbi.nlm.nih.gov/pmc/articles/PMC4870792/
Cryptophyta	http://rspb.royalsocietypublishing.org/content/early/2012/01/31/rspb.2011.2301<br>http://rspb.royalsocietypublishing.org/content/283/1823/20152802
Haptophyta	http://www.sciencedirect.com/science/article/pii/S187477871500029X<br>http://www.nature.com/nature/journal/v499/n7457/full/nature12221.html
