# Supplementary material for: Measuring the impact of gene prediction on gene loss estimates in Eukaryotes by quantifying falsely inferred absences
Source: PLoS Comput Biol. 2019 Aug 28;15(8):e1007301. doi: 10.1371/journal.pcbi.1007301 (PMC6736253; doi:10.1371/journal.pcbi.1007301)
Supplement: S2 Fig — Two Pfam domains previously inferred as absent are found in six frame translated DNA. Shown are the HMM overlapping with the scaffolds (x-axis) together with the bitscore. The hmmsearch tool “sequence output” is shown between the HMM and scaffold. (PDF) [file pcbi.1007301.s002.pdf]

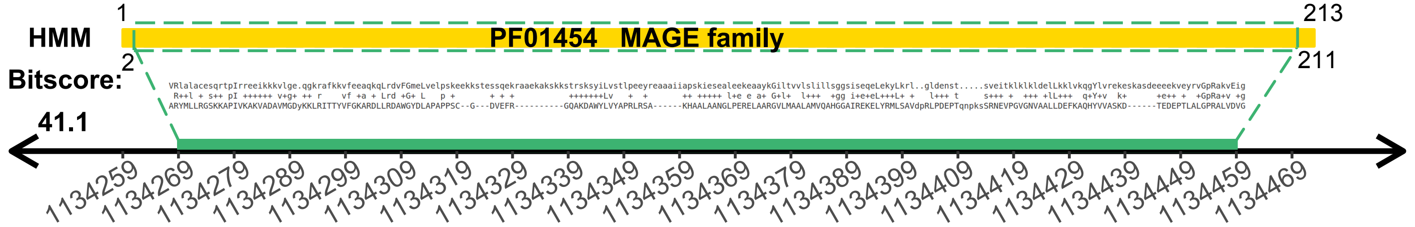

AANO − dna:supercontig supercontig:v_1_0:AURANscaffold_2:1:3465789:1 − frame 5

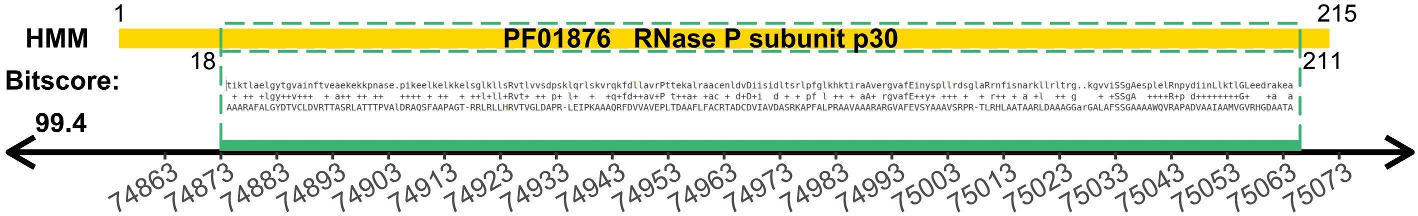

AANO − dna:supercontig supercontig:v_1_0:AURANscaffold_16:1:966663:1 − frame 2
